# Perception of the covid-19 vaccination by the medical profession

**DOI:** 10.1192/j.eurpsy.2023.1674

**Published:** 2023-07-19

**Authors:** H. Abrebak, F. Z. chamsi, A. Essafi, S. Radi, A. Taqui, A. El ammouri

**Affiliations:** 1 faculty of medicine of tangier; 2 faculté de médecine de tanger, Tangier, Morocco

## Abstract

**Introduction:**

By the end of October 2022, COVID-19 had infected more than 629 million people, with more than 6,5 million deaths worldwide.

During the pandemic, there have not been any specific antiviral drugs to effectively treat COVID-19, but non-specific drugs have been used and may improve the prognosis of high-risk patients with the disease. A vaccine is then considered the effective choice to stop this pandemic. The vaccine campaign against COVID-19 has been launched in Morocco since February 2021. This campaign has sparked great controversy over its effectiveness and safety in Morocco, as well as abroad, especially after the launch of the 3rd booster dose of the vaccine.

**Objectives:**

We sought to assess individual perceptions among the medical profession regarding vaccination against COVID-19 in Morocco and to determine preferences among this particular population in order to facilitate vaccination coverage.

**Methods:**

It is a descriptive and analytical cross-sectional study on doctors of different medical specialties and general medicine students in Morocco. Data collection was done through an anonymous self-administered questionnaire completed online. The measuring instruments used were a questionnaire containing 29 questions. In addition to socio-demographic questions (age, sex, household composition and employment), the rest of the questionnaire aims to examine Moroccan doctors’ perceptions on COVID-19 vaccination, attitudes, beliefs and knowledge about the vaccine. Some survey items were adapted from other similar surveys, while others were created by the research team of the psychiatry laboratory of the CHU of Tangier. The data is grouped and then analyzed by statistical software (SPSS v26).

**Results:**

There were 162 respondents with an average age of 26.52% with 66.7% of participants being female. 96% of doctors were vaccinated, 87.3% of them with 2 doses. 74% were vaccinated by Sinopharm, 23.5% by Astra Zeneca, 9.4% by Pfizer and 0.7% by Janssen. 68% had post-vaccination side effects. For unvaccinated doctors, 43% say they are not convinced of its usefulness, 28.5% find that there is not enough experience on its effectiveness and 14.3% report that they have a disease that contraindicates the vaccine.

For vaccinated doctors, 80% declare that they were vaccinated out of conviction and 20% out of obligation. 60% of our sample are against the obligation of the vaccination pass in public places, on the other hand 67% find that vaccination can stop the spread of the virus and 60% are ready to receive an annual vaccination if necessary.

**Conclusions:**

Physicians’ acceptance of the COVID-19 vaccine is important, as they are often a trusted source of vaccine information. Their vaccination can then positively influence the population, hence the need to integrate them into future awareness and prevention programs.

**Disclosure of Interest:**

None Declared

